# Reef fish hybridization: lessons learnt from butterflyfishes (genus *Chaetodon*)

**DOI:** 10.1002/ece3.83

**Published:** 2012-02

**Authors:** Stefano R Montanari, Lynne van Herwerden, Morgan S Pratchett, Jean-Paul A Hobbs, Anneli Fugedi

**Affiliations:** 1 School of Marine and Tropical Biology, James Cook UniversityTownsville, QLD 4811, Australia; 2 Molecular Ecology and Evolution Laboratory, James Cook UniversityTownsville, QLD 4811, Australia; 3 ARC Centre of Excellence for Coral Reef Studies, James Cook UniversityTownsville, QLD 4811, Australia

**Keywords:** Christmas island, discriminant analysis of principal components, ecological factors, evolutionary consequences, hybridization, hybrid zone, unidirectional hybridization

## Abstract

Natural hybridization is widespread among coral reef fishes. However, the ecological promoters and evolutionary consequences of reef fish hybridization have not been thoroughly evaluated. Butterflyfishes form a high number of hybrids and represent an appropriate group to investigate hybridization in reef fishes. This study provides a rare test of terrestrially derived hybridization theory in the marine environment by examining hybridization between *Chaetodon trifasciatus* and *C. lunulatus* at Christmas Island. Overlapping spatial and dietary ecologies enable heterospecific encounters. Nonassortative mating and local rarity of both parent species appear to permit heterospecific breeding pair formation. Microsatellite loci and mtDNA confirmed the status of hybrids, which displayed the lowest genetic diversity in the sample and used a reduced suite of resources, suggesting decreased adaptability. Maternal contribution to hybridization was unidirectional, and no introgression was detected, suggesting limited, localized evolutionary consequences of hybridization.

Comparisons to other reef fish hybridization studies revealed that different evolutionary consequences emerge, despite being promoted by similar factors, possibly due to the magnitude of genetic distance between hybridizing species. This study highlights the need for further enquiry aimed at evaluating the importance and long-term consequences of reef fish hybridization.

## Introduction

Natural hybridization occurs when individuals from different species or populations (distinguishable through one or more heritable characters) successfully interbreed, producing viable hybrids (Arnold 1997). To date, more than 25% of plant and 10% of animal species have been reported to hybridize in the wild (Mallet 2005). Certain taxonomic groups tend to hybridize much more than expected (Mallet 2005) and specific geographic regions concentrate hybridization in narrow “hybrid zones” (Barton and Hewitt 1985).

Of all the vertebrate groups, natural hybridization has most commonly been reported in fishes (Hubbs 1955; Allendorf and Waples 1996). More than 160 natural hybrids, involving 93 species across 12 families, have been reported among freshwater fishes (Scribner et al. 2000). In the marine environment 83 natural fish hybrids have been reported, involving 132 species across 17 families (S. Montanari, unpublished data). Where the freshwater literature is constellated with well-studied cases of natural fish hybridization across different climatic regions and levels of commercial interest, marine fish hybrids in the wild have received little attention and this has mainly been focused on commercially important, temperate water species (e.g., Norman 1934; Fujio 1977; Garcia de Leaniz and Verspoor 1989; Ruzzante et al. 2000; Nielsen et al. 2003; Ayllon et al. 2004; Burford et al. 2011). Only naturally occurring hybrids will be referred to as “hybrids” in this study.

Several processes have been proposed to explain the abundance of hybrid fishes, including: external fertilization (Hubbs 1955), competition for limited spawning grounds (Campton 1987), secondary contact of recently diverged sister taxa (McMillan and Palumbi 1995), spatial or dietary overlap in parental species (van Herwerden et al. 2006; Yaakub et al. 2006, 2007; Marie et al. 2007), rarity of one or both parental species (Gosline 1948; Randall et al. 1977; Frisch and van Herwerden 2006; Marie et al. 2007), sneak mating (van Herwerden et al. 2006), and absence of assortative mating (McMillan et al. 1999). Ecological observations of hybridizing reef fishes and comparison of these data to those collected from outside the hybrid zone—where available—may allow identification of ecological conditions that favor hybridization in the hybrid zone.

Genetic investigation of freshwater fish hybridization has revealed evolutionarily significant, though contrasting scenarios. For example, the explosive radiation (speciation) of cichlids in the African lakes has been attributed to protracted hybridization (Shaw et al. 2000; Turner et al. 2001; Verheyen et al. 2003; Seehausen 2004), and so has the collapse (reverse speciation) of the benthic and limnetic species of three-spined stickleback in the lakes of British Columbia (Taylor et al. 2006). The use of molecular genetics, clearly beneficial to understanding the evolutionary consequences of hybridization in freshwater fishes, has only recently been applied to reef fish hybridization (McMillan et al. 1999; van Herwerden et al. 2002, 2006; van Herwerden and Doherty 2006; Yaakub et al. 2006, 2007; Marie et al. 2007; Crow et al. 2010).

At least 75 species of coral reef fish species hybridize (Yaakub et al. 2006). Like most animal hybrids, reef fish hybrids have been traditionally identified through aberrant color patterns and morphological traits, often deemed intermediate between those of putative parent species (e.g., Randall 1956; Pyle and Randall 1994). This approach is still used today, and numerical predictions of hybrid color patterns (Miyazawa et al. 2010) as well as genetic validation of the hybrid status of intermediately colored individuals (e.g., Yaakub et al. 2006, 2007; Marie et al. 2007) have confirmed the soundness of this approach.

Prior reef fish hybridization studies have focused on members of the Acanthuridae (Marie et al. 2007), Chaetodontidae (McMillan et al. 1999), Labridae (Yaakub et al. 2006, 2007), Pomacentridae (van Herwerden and Doherty 2006), and Serranidae (van Herwerden et al. 2002, 2006; Frisch and van Herwerden 2006). Results from these studies have shown that reef fish hybridization can be characterized by unidirectional (e.g., Yaakub et al. 2006) or bidirectional (e.g., Marie et al. 2007) parental contributions, and the presence (e.g., van Herwerden et al. 2006) or absence (e.g., Yaakub et al. 2007) of introgression. Moreover, disparate ecological promoters of reef fish hybridization were identified, and varying levels of evolutionary significance ascribed to the process (McMillan et al. 1999; van Herwerden et al. 2002, 2006; Frisch and van Herwerden 2006; van Herwerden and Doherty 2006; Yaakub et al. 2006, 2007; Marie et al. 2007). Clearly, the combined use of ecological and genetic approaches in the study of reef fish hybridization can help elucidate the contribution of hybridization to the diversity and evolution of this group.

Butterflyfishes have the highest reported incidence of hybridization of all reef fish families (Allen et al. 1998; Kuiter 2002; Yaakub et al. 2006; Hobbs et al. In press): 44 of 130 (34%) species hybridize (Hobbs et al. In press), a proportion higher than most plant or animal taxa (Mallet 2005). The Chaetodontidae are a relatively young reef fish family (Bellwood et al. 2010), in which recently diverged allopatric sister species are common (Blum 1989). Many of these sister species have made secondary contact, setting the scene for hybridization (e.g., McMillan et al. 1999). Moreover, the dietary overlap shown by some species in this family (Pratchett 2005), together with habitat overlap, can increase the frequency of heterospecific encounters (favoring hybridization). In synergy, these characteristics of the Chaetodontidae render butterflyfish a suitable group for reef fish hybridization studies. Further, butterflyfishes are significantly affected by reef degradation (Pratchett et al. 2004, 2006b; Graham 2007), possibly due to the high incidence of corallivory in this group (Cole et al. 2008). Hybridization can result in increased adaptability to altered environments following disturbance (Grant and Grant 2002; Taylor et al. 2006; Riginos and Cunningham 2007) and may potentially be beneficial to butterflyfishes in a time when coral reefs are undergoing significant habitat changes.

Approximately 90% of hybridizing butterflyfishes occur at four specific geographical locations: southern Japan, Hawaii, Papua New Guinea-Micronesia, and the Eastern Indian Ocean (Hobbs et al. In press). Hybridizing reef fishes belonging to other families have been reported from these same locations (Pyle and Randall 1994; Gardner 1997; Kuriiwa et al. 2007; Hobbs et al. 2009). Christmas Island in the Eastern Indian Ocean is a known reef fish hybrid hotspot (Hobbs et al. 2009), where at least eight butterflyfish species hybridize (Hobbs et al. In press), making it an ideal location to study butterflyfish hybridization.

Prior studies of butterflyfish hybridization (McMillan et al. 1999) demonstrated that hybrid phenotypes largely outnumbered parental phenotypes within the hybrid zone, suggesting greater fitness of hybrids in the hybrid zone. Despite the wealth of reported butterflyfish hybrids, studies addressing the ecologies of these intermediate individuals have never been conducted. The results from McMillan et al. (1999) require that other butterflyfish hybrids be studied in the field, to determine whether adaptive disparities consistently result from hybridization among butterflyfishes. This study represents the most comprehensive examination of reef fish hybridization to date combining ecological, behavioral, and genetic approaches to investigate: (1) the ecology of hybridization between butterflyfishes *Chaetodon trifasciatus* Park 1797 and *C. lunulatus* Quoy and Gaimard 1824 at Christmas Island by assessing spatial and dietary overlap; (2) abundance of parental and hybrid individuals; (3) presence/absence of assortative pairing in parental species and their hybrids; (4) the directionality and evolutionary consequences of hybridization through molecular genetic analyses of mitochondrial and nuclear (microsatellite) DNA.

## Methods

### Ecology of hybridization

#### Study species

**Figure 1:**
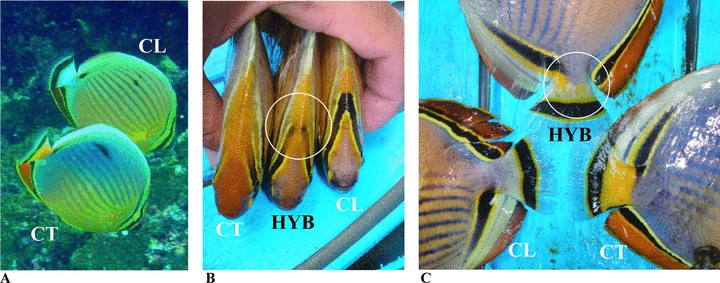
**(A)** *Chaetodon trifasciatus* (CT) and *C. lunulatus* (CL) swimming together in a heterospecific pair at Christmas Island; hybrids (HYB) of this species complex are characterized by their: **(B)** headband and **(C)** caudal peduncle, which are intermediate between those of their parents.

The Indian Ocean redfin butterflyfish, *C. trifasciatus* (Fig. 1A), ranges from East Africa to Bali, Indonesia, Cocos (Keeling), and Christmas Islands (Allen et al. 1998) at the easternmost periphery. The redfin butterflyfish, *C. lunulatus* (Fig. 1A), is widespread throughout the Western Pacific Ocean, from Eastern Australia north to Japan and east to Hawaii and the Tuamotu Islands (Allen et al. 1998). Christmas Island is at the westernmost edge of its distribution range (Allen et al. 1998; Hobbs and Salmond 2008), where heterospecific pairs are formed with *C. trifasciatus* (Hobbs et al. 2009). Both species generally form homospecific pairs throughout their range (Allen et al. 1998) and the time of pairing in *C. lunulatus*—an obligate monogamous species (Yabuta 1997)—corresponds with the onset of sexual maturity (Pratchett et al. 2006a), indicating a reproductive basis for pairing in this complex. Hybrid *C. trifasciatus*×*lunulatus* are identified based on coloration of their headband (Fig. 1B) and caudal peduncle (Fig. 1C), both intermediate between those of their parents.

#### Study location

**Figure 2:**
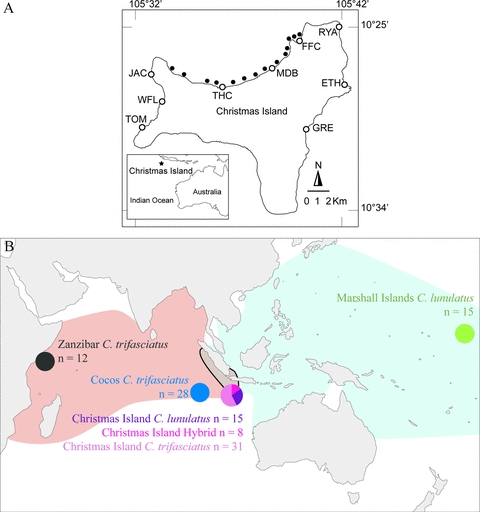
**(A)** Map of Christmas Island indicating the position of study sites used for the initial surveys (white dots) and the North coast surveys (black dots); Flying Fish Cove (FFC), Million Dollar Bombie (MDB), Thundercliff (THC), Jackson Point (JAC), Stef's Waterfall (WFL), Toms Point (TOM), Greta Beach (GRE), Ethel Beach (ETH), and Ryan's Ravine (RYA); inset shows the location of Christmas Island in the Indian Ocean. **(B)** Geographical origin and sample size (*n*) of populations of *Chaetodon trifasciatus*, *C. lunulatus,* and their hybrid used to assess phylogenetic relationships and population structure; the parental species ranges (redrawn from Allen et al. 1998) are also shown: *C. trifasciatus* (light red shaded area), *C. lunulatus* (light blue shaded area), and their contact zone (gray area with black outline).

This study was conducted in October 2010–November 2010 at Christmas Island, in the Indian Ocean (10°25′S–10°34′S, 105°32E–105°42E) (Fig. 2A, inset). Christmas Island is located approximately 360 km south of Java and is a recognized suture zone between Pacific and Indian Ocean taxa (Hobbs and Salmond 2008; Hobbs et al. 2009).

#### Island-wide survey

Underwater visual surveys were conducted at nine sites along the north, east, and west sides of the island (Fig. 2A). The South coast was inaccessible due to prevailing southeasterly winds. At each site, surveys were conducted at three depths: 5, 12, and 20 m, where six belt transects (50 × 5 m) were laid parallel to the depth contour, giving a total of 162 transects. On each transect, the number of each species and their hybrids was recorded. All individuals recorded during these preliminary surveys were encountered along the North coast and, accordingly, all subsequent sampling effort was concentrated there.

#### Ecological overlap

To assess habitat use of the parent species and hybrids, surveys were conducted along the North coast (Fig. 2A) to record the depth distributions of individuals in the complex. Approximately equal sampling was conducted at all depths from 3 to 25 m, reflecting the maximum depth range recorded for these species during prior reef-wide surveys. The specific depth at which individual fishes were first seen was recorded for 34 individuals of *C. trifasciatus,* 30 *C. lunulatus,* and eight putative hybrids. Depth distribution data were analyzed using an analysis of variance (ANOVA) comparing the mean depth at which each of the pure species and the putative hybrids were recorded.

To assess dietary overlap between parental species, as well as compare dietary composition of putative hybrids to that of parental species, in situ feeding observations were conducted for all individuals recorded during depth-based surveys. Three-minute observations were conducted for each individual following Pratchett (2005), recording the number of bites taken from different benthic prey or substrates. Prey items included scleractinian corals categorized based on genus and growth form as follows: *Montipora* (encrusting), *Porites* (encrusting or columnar), *Porites* (massive), *Acropora* (branching), *Acropora* (plate), *Acropora* (corymbose), *Galaxea* (encrusting), *Favites* (massive), *Pocillopora* (corymbose), *Echinopora* (foliose), *Lobophyllia* (massive), *Astreopora* (massive), *Diploastrea* (massive), *Fungia* (free living), other (encrusting), other (coral), and epilithic algal matrix (EAM).

Variation in dietary composition was analyzed using a multivariate analysis of variance (MANOVA), comparing the relative number of bites taken from each of the 17 prey categories by each species and the hybrid. To further test for similarities in feeding behavior, feeding rates (bites per 3 min) were compared between species and hybrids, using a one-way ANOVA.

#### Abundance

To obtain more precise estimates of abundance for rarer butterflyfish species, transect size was increased for additional North coast surveys (Thompson 2004). Abundances of all focal species were recorded at 14 sites along the North coast (Fig. 2A) while swimming along depth contours. The total area sampled for each replicate transect was calculated based on Global Positioning System (GPS) tracks. Recorded tracks were divided into deep and shallow transects which were independently measured, excluding the distances swum to reach the initial depth and the distance swum while moving between the deep and shallow waters. For the deep part of the dive (10–25 m), transect width was 10 m and average length was 323.56 m (average transect area 3235.64 m^2^). For the shallow part (3–9 m), the transect width was increased to 20 m (due to low abundances, and the increased width was appropriate given the high visibility and flat topography) and average length was 314.76 m (average transect area 6295.14 m^2^). Abundance data were analyzed using *t*-tests.

#### Assortative pairing

During large-scale abundance surveys, the composition of all pairs was recorded to assess whether pairing within this species complex is assortative or nonassortative. Partners were recorded for all individuals surveyed, regardless of whether the partner was encountered, and therefore counted, within the transect area. Fish not yet paired up were recorded as single individuals and most of these were of small size (< 100 mm total length) and probably juveniles. Expected pairing frequencies were calculated for each taxon by multiplying the total number of paired individuals by the proportional observed abundances of each of the three members of the complex. Pairing data were analyzed separately for each taxon in the complex using χ^2^-test between the expected and observed pairing frequencies.

### Genetics of hybridization

#### Sampling

Samples of *C. trifasciatus, C. lunulatus,* and hybrids were collected from within the hybrid zone at Christmas Island (but outside the survey locations), between 2005 and 2008 (Fig. 2B). Samples of the Indian Ocean species *C. trifasciatus* from outside the hybrid zone were collected at Cocos (Keeling) Islands (2005–2008) and Zanzibar (Fig. 2B). Similarly, *C. lunulatus* from outside the hybrid zone were collected from the Marshall Islands in the Pacific Ocean (2008–2009) (Fig. 2B). To obtain genetic samples, individual fishes were speared by scuba divers and, following capture, fin clips were immediately placed in 80% ethanol for subsequent analyses. The inclusion of samples of each “purebred” parental species from outside the hybrid zone allowed identification of species-specific genetic signals for comparison with the signal obtained at Christmas Island, where hybridization is apparent. Fin clips of *C. citrinellus* from Lizard Island, stored in 80% ethanol, were obtained from M. Pratchett and utilized as an outgroup taxon in all subsequent phylogenetic analyses (Fessler and Westneat 2007).

#### Laboratory procedures

For all subsequently described laboratory procedures and analyses, DNA was extracted from fin clips using a 5% Chelex extraction protocol (Walsh et al. 1991).

Approximately 600 bp of the mitochondrial gene cytochrome (cyt) b were amplified in both species and hybrids using cyt b specific primers (CBMP95–1; 5′-ATTCTAACTGGACTATTCCTTGCC-3′ and CBMP95–2; 5′-ATTATCTGGGTCTCCGAA(C/T)AGGTT-3′) previously utilized to study color pattern evolution in butterflyfishes of genus *Chaetodon* (McMillan and Palumbi 1995).

Polymerase chain reactions (PCR) were conducted as follows: twenty microliters reactions containing 2.5 mM Tris-Cl (pH 8.7), 5 mM KCl, 5 mM (NH_4_)_2_SO_4_, 200 µM each dNTP, 1.5–2 mM MgCl_2_, 0.25 µM each primer, 1 unit of Biotaq DNA polymerase (BIOLINE™), and 2 µl of Chelex extracted DNA template. Thermocycling was carried out with an initial denaturation step of 2 min at 94°C, followed by 35 cycles of denaturation, annealing, and extension (94°C for 30 sec, 50°C for 30 sec, 72°C for 90 sec) with a final extension of 10 min at 72°C. PCR products were visually confirmed using 1.5% agarose gel electrophoresis and amplicon sizes estimated with a 100-bp standard marker (BIOLINE™ Hyperladder IV).

PCR products were purified using either a standard isopropanol precipitation protocol (Sambrook et al. 1989) or a Sephadex G-25 resin 350-µl column spin protocol. Purified PCR products were sequenced with both primers using ABI (Applied Biosystems Incorporated) technologies either at Macrogen Seoul, South Korea or at the Australian Genome Research Facility (AGRF) Brisbane, Australia. GenBank accession numbers for all sequences are JQ012110 - JQ012216.

Sixteen *C. lunulatus* microsatellite markers (Lawton et al. 2010) were tested on two individuals each of *C. lunulatus* and *C. trifasciatus* and on three individuals of the putative hybrid. Detailed information about the primers used is presented in Table 1. PCR was conducted as described above, except primer concentration was increased to 0.5 µM. Thermocycling was carried out with an initial denaturation step of 3 min at 94°C, followed by a touchdown protocol of denaturation, annealing, and extension constituted of five cycles (94°C for 30 sec, primer-specific temperature (T_a_) °C(Table 1) for 30 sec, 72°C for 90 sec) plus 30 cycles (94°C for 30 sec, T_a_- 2°C for 30 sec, 72°C for 90 sec) and a final extension of 10 min at 72°C.

**Table 1:** Details of 16 microsatellite loci developed for *Chaetodon lunulatus* by Lawton et al. (2010). Primer sequences and repeat motifs are provided as given by the authors of the original study (Lawton et al. 2010). Annealing temperatures (T_a_) and size ranges are the ones utilized and found in the present investigation

Locus (label)	Primer sequence (5′-3′)	Repeat motif	T_a_ (°C)	Size range (bp)
Lun01 (FAM)	TGAACTGCAAAGCAACAACC	(CAT)_17_	55	2
	CTGCTTCTCTTTGGTGAGGAG			
Lun03 (TET)	TGTGTGTCACCACCTGGTCT	(AG)_29_	58	175–239
	ACTCAGTTTTGAGCCGCTTC			
Lun05 (FAM)	GCAACCCAGTCTCACATCAA	(CAA)_30_	55	155–191
	TCTGCTATTTCACAATTTTAGAGCA			
Lun07 (HEX)	AAGTGCCCTTTAGCAAAGCA	(TG)_17_	58	153–212
	CTCCAGTCGCTTTCTGTGTG			
Lun08 (HEX)	GGCCTTTGTTTGTGGTCATT	(CA)_26_	55	174–226
	CCTGAAGAGAGAGCTGCTCAA			
Lun09 (TET)	CCTGTGTTTGTCATCCAACG	(TG)_15_	58	143–167
	CTTTGGGACACACACTTCCA			
Lun10 (TET)	TTGTGTTGTTTTAGTGTTCCCTTT	(AC)_24_	58	223–285
	TGAGTGGTTATGATACATTAGATTTTG			
Lun14 (HEX)	TACGTTGGACAGTGGCTGTG	(TCA)_11_	58	207–240
	TGGCTCTGTGGCATGTATGT			
Lun17 (^*^)	TCAGAGGTCGCTAACGTGTG	(GAT)_12_	55	1
	CTCTAACGCGTCCTCTGTCC			
Lun19 (FAM)	TCCAGTTCCATTCTGCCTTT	(GAT)_16_	55	125–188
	CCGTCATTAACCTCCAGCAG			
Lun20 (TET)	CAGTGTCGGAGAACAACGAA	(CTT)_12_	58	2
	TCACTGTGTCACCAATGCAC			
Lun21 (FAM)	CAGGGAAAATCACACTTTCACA	(TGCC)_14_	55	223–283
	TGTCAAGCTGTGTGGGACAT			
Lun22 (HEX)	GGATGATGCAACTGATGGAA	(ATC)_15_	58	2
	TGTAGCATTTCATCTTTGACACTG			
Lun29 (HEX)	CACCCACAGGCAGTGTATTG	(AC)_33_	55	233–277
	GCCAGCCTGTCAAAACTTTA			
Lun34 (TET)	CATGCTTGGGTGAGCATGTA	(CA)_36_	58	165–185
	TGTGCGTTTGTGCAAGTGTA			
Lun36 (FAM)	GCGTTTGACTTCACGTTTCA	(GT)_30_	58	188–238
	TGCAAAACAACAACCTACGG			

1 Discarded prior to genotyping.

2 Could not be scored reliably.

PCR products were visually confirmed by 1.5% agarose gel electrophoresis and amplicon sizes estimated with a 100-bp standard marker (BIOLINE™ Hyperladder IV). All markers reliably amplified in all individuals and product sizes were consistent with expectations (Lawton et al. 2010).

In light of the successful testing, the best 15 markers were labeled with fluorescent tags (HEX, FAM, or TET) (Table 1). One marker (Lun17) was discarded during this step because it was more economical in both time and funds to process five sets of three loci. PCR cocktails were made as described above except the forward primers were substituted with their labeled equivalents. Thermocycling was performed as described above.

PCR products were purified by centrifugation through 350-µl Sephadex G-25 resin columns and subsequently inspected for quality via 1.5% agarose gel electrophoresis prior to genotyping. Genotypes were run on an Amersham Biosciences Megabase Capillary Sequencer with a 400 bp standard at the Advanced Analytical Centre (AAC) at James Cook University, Townsville, Australia. All genotypic data were deposited in the Dryad Repository: doi:10.5061/dryad.20fc5v4j

#### Data compilation

Nucleotide sequences were aligned using the ClustalW (Thompson et al. 1994) algorithm and manually edited in Geneious Pro v5.3.3 (Biomatters Ltd.). Microsatellite genotypes were scored using Fragment Profiler v1.2 (Amersham Biosciences) and subsequently edited using Genalex 6.1 (Peakall and Smouse 2006).

#### Phylogenetic analyses

Phylogenetic relationships were inferred in order to establish whether the two parental species had fixed sequence differences when considering samples from outside the hybrid zone, allowing the identification of clades representative of the genetically unique parental species. This was done using four approaches: neighbor-joining (NJ) (Saitou and Nei 1987) and maximum parsimony (MP) (Eck and Dayhoff 1966) algorithms, implemented in Mega 4 (Tamura et al. 2007), Bayesian inference (BI), run through the Mr. Bayes (Huelsenbeck and Ronquist 2001) plug-in (compiled by Marc Suchard and the Geneious Team) from within Geneious Pro v5.3.3 (Biomatters Ltd.), and maximum likelihood (ML) analysis, performed using Garli v0.95 (Zwickl 2006) and Bootscore v3.11 (Sukumaran 2007). In all analyses described here, trees were outgroup rooted with two individuals of *C. citrinellus*.

In the NJ algorithm, evolutionary distances were computed using the Maximum Composite Likelihood method (Tamura et al. 2004) with 1000 Bootstrap replicates. All gaps and missing data were pairwise deleted.

Ten independent MP analyses were run and the overall shortest tree selected. The tree was obtained using a Close-Neighbour-Interchange algorithm (Nei and Kumar 2000) with search level 2 (Eck and Dayhoff 1966; Nei and Kumar 2000) with initial trees inferred by random addition (10 replicates). All gaps and missing data were discarded.

BI analysis used the JC69 substitution model (Jukes and Cantor 1969)—selected using jModelTest v0.1.1 (Guindon and Gascuel 2003; Posada 2008)—and was run using Markov chain Monte Carlo (MCMC) simulations with four chains of 100,000 generations each, sampling trees every 100 generations. A 50% majority rule consensus tree was computed using the 1000 best post-burn-in trees.

ML analysis was repeated independently 10 times and the resulting best trees compared to ensure consistency of topology. A bootstrap ML analysis with 100 replicates was also run to compute a consensus tree based on the best topology previously obtained.

#### Population genetic analyses

A minimum spanning network (MSN) of cyt b haplotypes was constructed in Hapstar v0.6 (Prim 1957; Excoffier et al. 2005) and subsequently edited in Illustrator CS (Adobe Systems Inc.). Genetic diversity indices of cyt b haplotypes, including haplotype diversity (*h*) and nucleotide diversity (π), were calculated for all populations sampled using Arlequin v3.1 (Excoffier et al. 2005). Spatial heterogeneity for cyt b was assessed through analysis of molecular variance (AMOVA) and pairwise *F*_st_, performed in Arlequin with 1000 permutations.

Twelve of 15 markers genotyped were successfully scored, while three (Lun01, Lun20, and Lun22) could not be scored confidently and were therefore excluded from further analyses. Microsatellite metrics including number of alleles (N_a_), private alleles (P_a_), observed (H_O_), and expected (H_E_) heterozygosities and average inbreeding coefficient (*F*_IS_) were calculated in Genalex (Peakall and Smouse 2006) and Fstat v2.9 (Goudet 1995). Probabilities of departure from Hardy–Weinberg equilibrium (HWE) and linkage disequilibrium (LD) were estimated in Genepop v4.0 (Rousset 2008) using Markov chains with dememorization 10,000, 20 batches, and 5000 iterations per batch. The presence of null alleles, large allele dropout, and scoring bias were assessed using Microchecker v2.2.3 (van Oosterhout et al. 2004).

Raw estimates of population structure were calculated locus-by-locus and as an average over 11 loci (Lun34 was excluded due to >5% missing data) using AMOVA in Arlequin with 1000 permutations (Excoffier et al. 1992). Four individuals missing data at more than three loci were excluded from raw estimates of population differentiation, leaving a sample (*n* = 105) in which all individuals had at least eight loci and each locus <5% missing data.

Null allele frequencies and associated Excluding Null Alleles (ENA) corrected estimates of population structure were calculated in Freena (Chapuis and Estoup 2007). In this analysis, missing data were regarded as null homozygotes. Estimators of actual differentiation (D_est_), which have been indicated as particularly suitable in estimating population structure in the presence of high heterozygosities and small sample size (Jost 2008), were also calculated, using the web-based algorithm Smogd v1.2.5 (Crawford 2010).

To further investigate population structures inferred through conventional analytical approaches, a discriminant analysis of principal components (DAPC) (Jombart et al. 2010) was run on all 12 microsatellite loci. This multivariate method is designed to extract information from genetic datasets and assign genotypes to predefined clusters (Jombart et al. 2010). In DAPC, a linear discriminant analysis (DA) is conducted on genotypic information, previously transformed into uncorrelated components through a principal component analysis (PCA) (Jombart et al. 2010). In doing so, the shortcomings of both PCA and DA in their applicability to genotypic datasets are overcome (Jombart et al. 2010). This method allows the retention of a significant proportion of the genetic variability while it minimizes within- and maximizes between-population variance (Jombart et al. 2010). Furthermore, DAPC is robust to deviations from HWE and LD and is as sensitive—but not as computationally intensive—as Bayesian clustering approaches (Jombart et al. 2010). In DAPC, the tradeoff between obtaining stable results and explaining most genetic variability is still under debate (T. Jombart, pers. comm.). In the present analysis, 73 PCs were retained in the DA, accounting for 90% of the genotypic variability. DAPC was implemented in R v2.12 (http://www.Rproject.org) using functions *dudi.pca* and *dapc* from the R packages ade4, adegenet, and MASS. The results were visualized in a scatterplot generated by adegenet (Jombart 2008; Jombart et al. 2010).

## Results

### Ecology of hybridization

**Figure 3:**
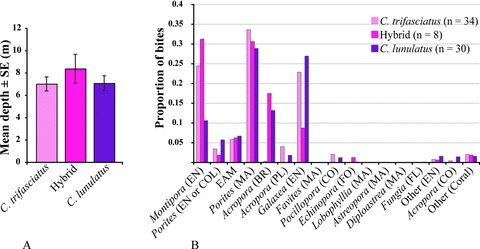
**(A)** Mean depth distribution (± SE) of *C. trifasciatus* (*n* = 34), *C. lunulatus* (*n* = 30), and hybrid (*n* = 8) on the North coast of Christmas Island. **(B)** Proportional dietary composition of the parental species and hybrid at Christmas Island. Data are based on direct 3-min feeding observations of each species and the hybrid. Coral food categories are grouped by genus and growth form: encrusting (EN), columnar (COL), massive (MA), branching (BR), plate (PL), corymbose (CO), foliose (FO), and free living (FL). The category other (EN) includes encrusting corals not belonging to the specified encrusting genera. The category other (Coral) includes all other, nonencrusting coral.

*Chaetodon trifasciatus* and *C. lunulatus* have very similar ecologies, reflected in strongly overlapping patterns of abundance and dietary composition. *Chaetodon trifasciatus* (average depth 7 m ± 0.62 SE) and *C. lunulatus* (average depth 7.1 m ± 0.66 SE) occupied relatively narrow, largely overlapping depth ranges (4–15 m and 3–18 m, respectively) and were most frequently (70% and 67% of individuals, respectively) encountered at 5–8 m depth (Fig. 3A). There was no significant difference in the depth distributions of *C. trifasciatus* (*n* = 34) and *C. lunulatus* (*n* = 30) (ANOVA, df = 1, *F* = 0.006, *P* = 0.94) (Fig. 3A). While hybrids tended to be found slightly deeper than the parent species (8.4 m ± 1.3 SE), the depth distribution of hybrids (*n* = 8) did not significantly differ from those of their parents (ANOVA, df = 2, *F* = 0.489, *P* = 0.615) (Fig. 3A).

*Chaetodon trifasciatus* (*n* = 34) and *C. lunulatus* (*n* = 30) each fed on a broad range (11 and 13, out of 17, respectively) of prey items, and there was no significant difference in the relative use of these preys (MANOVA, Pillai's Trace = 0.715, hypothesis df = 32, *P* = 0.062) (Fig. 3B). Over 50% of the diet of *C. trifasciatus* and *C. lunulatus* was constituted of massive *Porites* and encrusting *Galaxea* corals (Fig. 3B). *Chaetodon trifasciatus* and *C. lunulatus* also frequently consumed encrusting *Montipora* corals (25% and 10%, respectively). The hybrids (*n* = 8) were observed feeding mostly on encrusting *Montipora* (> 30%) and massive *Porites* (> 30%), and utilized nine of 17 food categories (eight corals and EAM) (Fig. 3B). Other major coral genera consumed by the hybrids included branching *Acropora* (> 15%), *Galaxea* (< 10%), and *Echinopora* (< 5%).

The feeding rates of both species and their hybrid were also comparable. *Chaetodon trifasciatus* took an average of 29 (± 2.8 SE) bites over 3 min, *C. lunulatus* 24 (± 2.1 SE), and the hybrids 36 (± 5 SE) bites (ANOVA, df = 2, *F* = 2.436, *P* = 0.09). Furthermore, both species and the hybrid consumed an average of four (± 0.29 SE, ± 0.26 SE, ± 0.18 SE, respectively) prey types over 3 min. Moreover, when observed in mixed pairs, individuals of different species were often feeding on the same prey in largely overlapping areas.

#### Abundance

**Figure 4:**
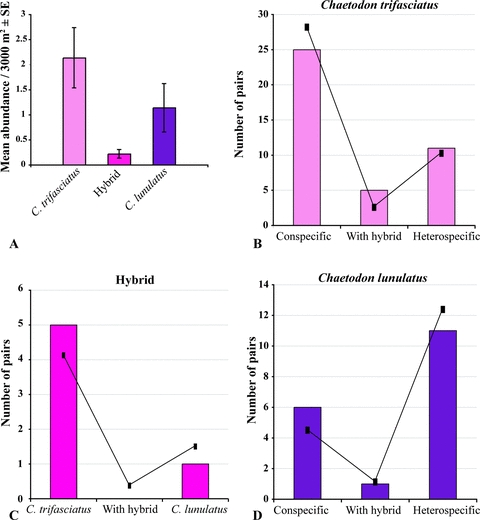
(A) Mean abundance (per 3000 m^2^± SE) of the parental species and hybrid along the North coast of Christmas Island. (B), (C), and (D) Expected (lines) and observed (bars) pairing frequencies of (B) *C. trifasciatus*, (C) hybrids, and (D) *C. lunulatus* at Christmas Island. Expected frequencies were calculated based on observed abundances of paired individuals.

Underwater visual surveys indicated both parent species were relatively uncommon, and there was no significant difference in abundance of the parental species (*t*-test, *t* = 1.285, df = 38, *P* = 0.207) (Fig. 4A). The average abundance (individuals per 3000 m^2^) of *C. trifasciatus* was two (± 0.60 SE) and *C. lunulatus* was one (± 0.48 SE) (Fig. 4A). Hybrids were even less abundant than their parents (*t*-test, *t* = 3.552, df = 42, *P* = 0.001) (Fig. 4A). The average density of hybrids was approximately one individual per 12,000 m^2^ (Fig. 4A). Overall, only eight hybrids were encountered in all surveys—which covered >170,000 m^2^ of reef habitat at Christmas Island.

#### Assortative pairing

The pairing frequencies of both species and the hybrids did not differ significantly from expectations based on relative abundances (*C. trifasciatus*: χ^2^ = 2.73, df = 2, *P* = 0.26; *C. lunulatus*: χ^2^ = 0.67, df = 2, *P* = 0.71; Hybrids: χ^2^ = 0.73, df = 2, *P* = 0.69) (Figs. 4B–D), indicating that members of this complex pair nonassortatively. *Chaetodon trifasciatus* (paired individuals, *n* = 41) paired conspecifically in more than 60% of cases, heterospecifically with *C. lunulatus* in almost 27% and with hybrids in 12% of pairs (Fig. 4B).

*Chaetodon lunulatus* (paired individuals, *n* = 18) paired conspecifically in 33% of cases, more frequently with *C. trifasciatus* (61%) and with hybrids in 5% of pairs (Fig. 4D). The hybrids (paired individuals, *n* = 6) were never observed together in a pair, but formed pairs with both parents, most frequently with *C. trifasciatus* (83%) (Fig. 4C).

### Genetics of hybridization

**Figure 5:**
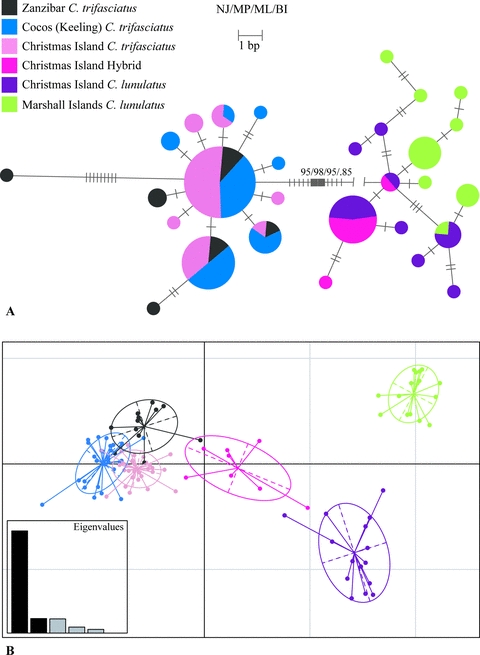
**(A)** Scaled branch length minimum spanning network (MSN) depicting the genealogical relationships between haplotypes in the *Chaetodon trifasciatus* complex. Haplotypes are represented by scaled circles showing the origin and number of individuals sharing a haplotype. Branches are to scale with number of substitutions (bp) and perpendicular bars on branches are substitution counts (thin line = 1 substitution; thick line = 10 substitutions). The branch separating the two clades has been truncated for ease of representation. Bootstrap support values for phylogenetic relationships inferred by neighbor-joining (NJ), maximum parsimony (MP) and maximum likelihood (ML), and posterior probabilities from Bayesian Inference of phylogeny (BI) are shown along the main branch. **(B)** Scatterplot of the discriminant analysis of principal components (DAPC) (Jombart et al. 2010) performed on 12 microsatellite loci for six populations of the *C. trifasciatus* complex. Taxon and geographical origin of each population are indicated in the legend and depicted in the plot by colors and 95% inertia ellipses. Individual genotypes are represented by dots. The *x* and *y* axes represent the first two discriminant functions, respectively. The plot of eigenvalues shows the amount of genetic information retained by each successive discriminant function.

Five hundred and fifty-two basepair of the mitochondrial cyt b region were resolved for a total of 105 individuals in the complex (details provided in Table 2). Of the 522 bp sequenced, 114 parsimony informative sites and 29 individual haplotypes were identified (Fig. 5A).

**Table 2:** Sample sizes (total *n* = 105) for cyt b analyses, number of haplotypes (nh), haplotype diversities (*h*), nucleotide diversities (π) of cyt *b* for all populations in the species complex

Population	n	nh	*h*	π
Zanzibar *C. trifasciatus*	10	6	0.89	0.007
Cocos Is. *C. trifasciatus*	28	10	0.82	0.002
Christmas Is. *C. trifasciatus*	28	8	0.71	0.001
Christmas Is. Hybrid	9	4	0.58	0.001
Christmas Is. *C. lunulatus*	15	8	0.83	0.005
Marshall Is. *C. lunulatus*	15	8	0.83	0.006

Summary statistics for 12 microsatellite loci are presented in Table 3. Hybrids showed the lowest allelic diversity and Christmas Island *C. trifasciatus* the highest. Significant single-locus departures from HWE were detected in 12 of 72 tests at population level before sequential Bonferroni correction and nine afterwards (α = 0.0083) (Table 3). Null alleles might contribute to departures from HWE in loci Lun10, Lun29, and Lun36. *Chaetodon trifasciatus* populations of Christmas and Cocos (Keeling) Islands had the highest number of private alleles (13 and 10, respectively), while hybrids had none (Table 3).

**Table 3:** Summary statistics for 12 microsatellite loci used in population genetic analyses. Sample sizes (*n*), observed number of alleles (*N*_a_), the average inbreeding coefficient (F_IS_), observed number of private alleles (P_a_), observed heterozygosity (H_O_), expected heterzygosity (H_E_), and probability of departure from HWE for each locus at each population (p)

Locus	Christmas Island *C. lunulatus* (n = 15)	Christmas Island Hybrids (n = 8)	Christmas Island *C. trifasciatus* (n = 31)	Marshall Islands *C. lunulatus* (n = 15)	Cocos (Keeling) Islands *C. trifasciatus* (n = 28)	Zanzibar *C. trifasciatus* (n = 12)
Lun03	*N*_a_ = 13	*N*_a_ = 13	*N*_a_ = 21	*N*_a_ = 11	*N*_a_ = 19	*N*_a_ = 15
	P_a_ = 1	P_a_ = 0	P_a_ = 3	P_a_ = 0	P_a_ = 4	P_a_ = 1
	H_O_ = 0.867	H_O_ = 0.875	H_O_ = 0.964	H_O_ = 0.733	H_O_ = 0.821	H_O_ = 0.750
	H_E_ = 0.818	H_E_ = 0.914	H_E_ = 0.940	H_E_ = 0.891	H_E_ = 0.907	H_E_ = 0.913
	F_IS_ = 0.057	F_IS_ = 0.109	F_IS_ = –0.008	F_IS_ = 0.210	F_IS_ = 0.112	F_IS_ = 0.220
	*P* = 0.132	*P* = 0.178	*P* = 0.426	*P* = 0.169	*P* = 0.112	*P* = 0.01*
Lun05	*N*_a_ = 9	N_a_ = 7	*N*_a_ = 8	*N*_a_ = 6	*N*_a_ = 9	*N*_a_ = 8
	P_a_ = 0	P_a_ = 0	P_a_ = 1	P_a_ = 0	P_a_ = 0	P_a_ = 0
	H_O_ = 0.857	H_O_ = 0.875	H_O_ = 0.815	H_O_ = 0.867	H_O_ = 0.786	H_O_ = 0.833
	H_E_ = 0.839	H_E_ = 0.836	H_E_ = 0.774	H_E_ = 0.807	H_E_ = 0.810	H_E_ = 0.802
	F_IS_ = 0.016	F_IS_ = 0.020	F_IS_ = –0.033	F_IS_ = –0.040	F_IS_ = 0.048	F_IS_ = 0.005
	*P* = 0.670	*P* = 0.836	*P* = 0.003**	*P* = 0.698	*P* = 0.097	*P* = 0.705
Lun07	*N*_a_ = 11	*N*_a_ = 12	*N*_a_ = 22	*N*_a_ = 12	*N*_a_ = 18	*N*_a_ = 14
	P_a_ = 0	P_a_ = 0	P_a_ = 1	P_a_ = 1	P_a_ = 0	P_a_ = 0
	H_O_ = 0.667	H_O_ = 1.000	H_O_ = 0.806	H_O_ = 1.000	H_O_ = 0.929	H_O_ = 1.000
	H_E_ = 0.818	H_E_ = 0.898	H_E_ = 0.932	H_E_ = 0.876	H_E_ = 0.927	H_E_ = 0.910
	F_IS_ = 0.218	F_IS_ = –0.047	F_IS_ = 0.151	F_IS_ = –0.108	F_IS_ = 0.017	F_IS_ = –0.056
	*P* = 0.000**	*P* = 1.000	*P* = 0.17	*P* = 0.855	*P* = 0.045*	*P* = 0.627
Lun08	*N*_a_ = 14	*N*_a_ = 8	*N*_a_ = 15	*N*_a_ = 14	*N*_a_ = 16	*N*_a_ = 15
	P_a_ = 0	P_a_ = 0	P_a_ = 0	P_a_ = 1	P_a_ = 1	P_a_ = 1
	H_O_ = 0.867	H_O_ = 0.750	H_O_ = 0.929	H_O_ = 1.000	H_O_ = 1.000	H_O_ = 0.917
	H_E_ = 0.902	H_E_ = 0.844	H_E_ = 0.913	H_E_ = 0.900	H_E_ = 0.917	H_E_ = 0.899
	F_IS_ = 0.074	F_IS_ = 0.176	F_IS_ = 0.001	F_IS_ = –0.077	F_IS_ = –0.071	F_IS_ = 0.024
	*P* = 0.259	*P* = 0.346	*P* = 0.235	*P* = 0.267	*P* = 0.961	*P* = 0.733
Lun09	*N*_a_ = 2	*N*_a_ = 3	*N*_a_ = 8	*N*_a_ = 3	*N*_a_ = 7	*N*_a_ = 3
	P_a_ = 0	P_a_ = 0	P_a_ = 2	P_a_ = 1	P_a_ = 1	P_a_ = 0
	H_O_ = 0.286	H_O_ = 0.875	H_O_ = 0.600	H_O_ = 0.533	H_O_ = 0.536	H_O_ = 0.333
	H_E_ = 0.245	H_E_ = 0.633	H_E_ = 0.571	H_E_ = 0.504	H_E_ = 0.605	H_E_ = 0.288
	F_IS_ = –0.130	F_IS_ = –0.324	F_IS_ = –0.034	F_IS_ = –0.023	F_IS_ = 0.132	F_IS_ = –0.114
	*P* = 1.000	*P* = 0.608	*P* = 0.742	*P* = 1.000	*P* = 0.037*	*P* = 1.000
Lun10	*N*_a_ = 12	*N*_a_ = 6	*N*_a_ = 13	*N*_a_ = 15	*N*_a_ = 15	*N*_a_ = 12
	P_a_ = 0	P_a_ = 0	P_a_ = 0	P_a_ = 3	P_a_ = 4	P_a_ = 2
	H_O_ = 0.786	H_O_ = 0.571	H_O_ = 0.393	H_O_ = 1.000	H_O_ = 0.480	H_O_ = 0.500
	H_E_ = 0.890	H_E_ = 0.776	H_E_ = 0.873	H_E_ = 0.907	H_E_ = 0.840	H_E_ = 0.861
	F_IS_ = 0.154	F_IS_ = 0.333	F_IS_ = 0.563	F_IS_ = –0.069	F_IS_ = 0.445	F_IS_ = 0.455
	*P* = 0.297	*P* = 0.242	*P* = 0.000**	*P* = 0.870	*P* = 0.000**	*P* = 0.000**
Lun14	*N*_a_ = 5	*N*_a_ = 3	*N*_a_ = 5	*N*_a_ = 2	*N*_a_ = 5	*N*_a_ = 4
	P_a_ = 2	P_a_ = 0	P_a_ = 0	P_a_ = 0	P_a_ = 0	P_a_ = 0
	H_O_ = 0.533	H_O_ = 0.750	H_O_ = 0.571	H_O_ = 0.400	H_O_ = 0.571	H_O_ = 0.455
	H_E_ = 0.538	H_E_ = 0.508	H_E_ = 0.551	H_E_ = 0.391	H_E_ = 0.591	H_E_ = 0.442
	F_IS_ = 0.043	F_IS_ = –0.424	F_IS_ = –0.019	F_IS_ = 0.012	F_IS_ = 0.052	F_IS_ = 0.020
	*P* = 0.681	*P* = 0.627	*P* = 0.520	*P* = 1.000	*P* = 0.336	*P* = 0.405
Lun19	*N*_a_ = 11	*N*_a_ = 11	*N*_a_ = 15	*N*_a_ = 10	*N*_a_ = 13	*N*_a_ = 10
	P_a_ = 1	P_a_ = 0	P_a_ = 0	P_a_ = 0	P_a_ = 0	P_a_ = 0
	H_O_ = 0.800	H_O_ = 0.875	H_O_ = 0.871	H_O_ = 0.867	H_O_ = 0.893	H_O_ = 0.818
	H_E_ = 0.887	H_E_ = 0.875	H_E_ = 0.887	H_E_ = 0.864	H_E_ = 0.902	H_E_ = 0.851
	F_IS_ = 0.132	F_IS_ = 0.067	F_IS_ = 0.035	F_IS_ = 0.032	F_IS_ = 0.028	F_IS_ = 0.086
	*P* = 0.192	*P* = 0.576	*P* = 0.455	*P* = 0.497	*P* = 0.014*	*P* = 0.321
Lun21	*N*_a_ = 14	*N*_a_ = 10	*N*_a_ = 17	*N*_a_ = 11	*N*_a_ = 13	*N*_a_ = 11
	P_a_ = 0	P_a_ = 0	P_a_ = 2	P_a_ = 1	P_a_ = 0	P_a_ = 1
	H_O_ = 0.929	H_O_ = 1.000	H_O_ = 0.929	H_O_ = 0.800	H_O_ = 0.889	H_O_ = 0.917
	H_E_ = 0.908	H_E_ = 0.844	H_E_ = 0.899	H_E_ = 0.860	H_E_ = 0.905	H_E_ = 0.878
	F_IS_ = 0.015	F_IS_ = –0.120	F_IS_ = –0.015	F_IS_ = 0.104	F_IS_ = 0.037	F_IS_ = 0.000
	*P* = 0.413	*P* = 0.800	*P* = 0.777	*P* = 0.278	*P* = 0.372	*P* = 0.622
Lun29	*N*_a_ = 8	*N*_a_ = 10	*N*_a_ = 20	*N*_a_ = 13	*N*_a_ = 18	*N*_a_ = 13
	P_a_ = 0	P_a_ = 0	P_a_ = 0	P_a_ = 1	P_a_ = 0	P_a_ = 0
	H_O_ = 0.857	H_O_ = 0.875	H_O_ = 0.714	H_O_ = 1.000	H_O_ = 0.704	H_O_ = 0.833
	H_E_ = 0.821	H_E_ = 0.859	H_E_ = 0.932	H_E_ = 0.884	H_E_ = 0.931	H_E_ = 0.906
	F_IS_ = –0.006	F_IS_ = 0.049	F_IS_ = 0.251	F_IS_ = –0.097	F_IS_ = 0.262	F_IS_ = 0.124
	*P* = 0.438	*P* = 0.734	*P* = 0.001**	*P* = 0.480	*P* = 0.001**	*P* = 0.282
Lun34	*N*_a_ = 3	*N*_a_ = 6	*N*_a_ = 9	*N*_a_ = 3	*N*_a_ = 7	*N*_a_ = 8
	P_a_ = 0	P_a_ = 0	P_a_ = 2	P_a_ = 0	P_a_ = 0	P_a_ = 1
	H_O_ = 0.600	H_O_ = 0.875	H_O_ = 0.840	H_O_ = 0.467	H_O_ = 0.833	H_O_ = 0.909
	H_E_ = 0.558	H_E_ = 0.750	H_E_ = 0.821	H_E_ = 0.451	H_E_ = 0.809	H_E_ = 0.814
	F_IS_ = –0.041	F_IS_ = –0.101	F_IS_ = –0.003	F_IS_ = 0.000	F_IS_ = –0.009	F_IS_ = –0.070
	*P* = 0.632	*P* = 0.707	*P* = 0.09	*P* = 1.000	*P* = 0.386	*P* = 0.727
Lun36	*N*_a_ = 17	*N*_a_ = 7	*N*_a_ = 15	*N*_a_ = 15	*N*_a_ = 9	*N*_a_ = 8
	P_a_ = 1	P_a_ = 0	P_a_ = 2	P_a_ = 0	P_a_ = 0	P_a_ = 0
	H_O_ = 0.933	H_O_ = 0.625	H_O_ = 0.621	H_O_ = 0.600	H_O_ = 0.667	H_O_ = 0.750
	H_E_ = 0.929	H_E_ = 0.648	H_E_ = 0.705	H_E_ = 0.916	H_E_ = 0.580	H_E_ = 0.663
	F_IS_ = 0.030	F_IS_ = 0.103	F_IS_ = 0.136	F_IS_ = 0.375	F_IS_ = –0.130	F_IS_ = –0.088
	*P* = 0.693	*P* = 0.454	*P* = 0.008**	*P* = 0.000**	*P* = 0.837	*P* = 0.971

* *P* < 0.05

** *P* < 0.0083 after sequential Bonferroni correction (highlighted in bold).

#### Phylogenetic analyses

Phylogenetic relationships were inferred using four methods, which all produced highly congruent tree topologies. The bootstrap support values from NJ, MP, ML, and the posterior probabilities from BI are reported on the scaled branch MSN depicting genealogical relationships between haplotypes (Fig. 5A). The main partition was well supported across all analyses, identifying a clear separation between the two parental clades (*C. trifasciatus* and *C. lunulatus*) with 29 fixed nucleotide changes separating the species (5% divergence at cyt b). Hybrids shared haplotypes with the Pacific Ocean *C. lunulatus* parental clade only, indicating a unidirectional maternal contribution to hybridization (Fig. 5A). No evidence of introgression was found between parental species in this complex (Fig. 5A).

#### Population genetic analyses

Molecular diversity indices for mitochondrial cyt b are presented in Table 2. The Zanzibar population of *C. trifasciatus* had the highest haplotype (*h*) and nucleotide (π) diversities, *C. lunulatus* from within the Christmas Island hybrid zone had the second highest *h* and third highest π (Table 2). For the Indian Ocean *C. trifasciatus, h* and π were lower as geographic distance from the hybrid zone decreased, and lowest at Christmas Island (Table 2). Conversely, the Pacific Ocean sister species, *C. lunulatus*, had the lowest π in the population furthest away from the hybrid zone—but the two populations had same *h* (Table 2). Hybrids had the lowest π and the second lowest *h* of all populations in this complex (Table 2).

The level of genetic population differentiation was high for all comparisons (Tables 4, 5). The AMOVA fixation index for the mitochondrial cyt b marker was Φ_st_ = 0.905, *P* < 0.0001. The microsatellite data also revealed a high level of population structure (raw Φ_st_ = 0.055, *P* < 0.0001; D_est_ = 0.194 *P* < 0.0001) and raw values were comparable to those corrected for null alleles (Table 5). For cyt b and the 12 microsatellite loci, nearly all pairwise comparisons were significant (F_st_ and D_est_, respectively) (Table 4). Not surprisingly, there was clear genetic structuring between the parental species (Table 4). Conversely, cyt b analyses failed to detect significant population structure between *C. trifasciatus* samples from Christmas and Cocos (Keeling) Islands (Table 4A). The microsatellite data, in this respect, confirm the lack of structure between these two populations, and indicate that such is the case for *C. trifasciatus* populations across the whole Indian Ocean, including Zanzibar (Table 4B). Populations of *C. lunulatus* from separate ocean basins showed significant population structure, according to cyt b and microsatellite data (Table 4). The hybrid population significantly differed from all other populations (Table 4). Also to note is the clear structure in both mitochondrial and nuclear data between the parental populations from within the hybrid zone (Table 4), despite the apparent hybridization—indicating lack of introgression.

**Table 4:** Pairwise population comparisons: (**A**) F_st_ generated from 552 bp of mitochondrial cyt b gene (hybrid population *N* = 9 was omitted due to sample size); (**B**) estimator of actual differentiation (D_est_) (Jost 2008) based on 12 microsatellite loci and corresponding *P* values (upper diagonal)

	CL (CI)	CL (RMI)	HYB (CI)	CT (CK)	CT (CI)	CT (Z)
A						
CL (CI)		0.009	0.017	<0.001	<0.001	<0.001
CL (RMI)	0.168		0.006	<0.001	<0.001	<0.001
HYB (CI)	0.190	0.284		<0.001	<0.001	<0.001
CT (CK)	0.945	0.958	0.968		0.428	0.037
CT (CI)	0.950	0.963	0.975	0.000		0.015
CT (Z)	0.901	0.922	0.931	0.076	0.099	
B						
CL (CI)		0.018	<0.001	<0.001	<0.001	<0.001
CL (RMI)	0.005		<0.001	<0.001	<0.001	<0.001
HYB (CI)	0.096	0.121		<0.001	0.009	<0.001
CT (CK)	0.328	0.375	0.050		0.135	0.117
CT (CI)	0.301	0.363	0.047	0.016		0.694
CT (Z)	0.289	0.241	0.011	0.003	0.000	

Significant comparisons are highlighted in bold.

CL = *C. lunulatus*; CT = *C. trifasciatus* and HYB = hybrids; CI = Christmas Island; CK = Cocos (Keeling) Islands; RMI = Marshall Islands and Z = Zanzibar.

**Table 5:** Raw population differentiation from microsatellite allele frequencies , population differentiation corrected for null allele frequencies using the ENA correction of Chapuis and Estoup (2007), and estimator of actual differentiation (Jost 2008) (D_est_); results are presented locus-by-locus and as an average over 12 loci. All values are significant to the 95% confidence interval

Locus	Raw	ENA corrected	D_est_
Lun05	0.033	0.037	0.161
Lun08	0.009	0.008	0.112
Lun09	0.298	0.296	0.472
Lun21	0.010	0.009	0.115
Lun14	0.048	0.049	0.052
Lun19	0.017	0.016	0.164
Lun34	0.096	0.100	0.291
Lun10	0.053	0.033	0.475
Lun29	0.021	0.023	0.294
Lun03	0.017	0.016	0.258
Lun07	0.035	0.032	0.374
Lun36	0.080	0.081	0.260
Average over 12 loci	0.055^1^	0.054	0.194

1 11 loci only: locus Lun34 was excluded due to >5% missing data.

The clear genetic population structuring shown by traditional analyses of both mtDNA and nuclear DNA was also visible in the DAPC of 12 microsatellite loci (Fig. 5B). The two parental species were clearly clustered within species and separated from each other regardless of sampling location (Fig. 5B). Populations of *C. lunulatus—*Christmas and Marshall Islands—sampled from separate ocean basins showed clear separation, while populations of *C. trifasciatus* from the Indian Ocean—as far apart as Christmas Island and Zanzibar—were much less clearly separated irrespective of geographical distance (Fig. 5B). The hybrid population was distinct from all others, and genotypes of this population were intermediate between those of the parental species (Fig. 5B).

## Discussion

This study genetically confirmed hybridization between the butterflyfishes *C. trifasciatus* and *C. lunulatus* at Christmas Island, determined the evolutionary consequences of this process and identified ecological conditions that favor hybridization. Direct field observations suggest that low abundances of both species and nonassortative mating may have promoted interbreeding. Furthermore, genetic analyses of mtDNA and microsatellite loci have confirmed that distinct and intermediate color morphs are hybrids, and shown that there is a unidirectional maternal contribution to hybridization and no introgression between the parental species. These findings suggest that the production of viable hybrids between *C. trifasciatus* and *C. lunulatus* is relatively rare. Apparently hybridization may facilitate persistence of the Pacific Ocean species at Christmas Island, but this may be at the expense of the Indian Ocean species at this isolated location.

### Ecology of hybridization

The recently diverged, sister species *C. trifasciatus* and *C. lunulatus* (Bellwood et al. 2010) have largely allopatric distributions (Allen et al. 1998) and occur in sympatry at Christmas Island, where they form heterospecific pairs. In butterflyfishes, cases of hybridization between allopatric sister species that come into secondary contact, such as this one, represent the minority—13 of 35 (37%) (Hobbs et al. In press). Sympatry of Indian and Western Pacific Ocean species at Christmas Island has been considered a potential indication of westward dispersal of Pacific Ocean taxa (Allen and Steene 1979; Blum 1989), has set the scene for previously reported cases of reef fish hybridization (Marie et al. 2007; Hobbs et al. 2009) and is a precursor for hybridization between *C. trifasciatus* and *C. lunulatus*.

At Christmas Island, *C. trifasciatus* and *C. lunulatus* have very similar ecologies (dietary composition and habitat use), which increases the chance of heterospecific encounters. Ecological similarities and habitat overlap among recently diverged species are not uncommon, and act to increase (or at least do not limit) social interactions between hybridizing reef fishes (Randall 1956; Fischer 1980; Frisch and van Herwerden 2006; Yaakub et al. 2006, 2007; Marie et al. 2007). Both *C. trifasciatus* and *C. lunulatus* are known obligate corallivores (Harmelin–Vivien 1989; Pratchett et al. 2004; Berumen et al. 2005; Pratchett 2005; Cole et al. 2008) and accordingly, at Christmas Island, both species feed almost exclusively on scleractinian corals, mostly *Porites*, *Galaxea,* and *Montipora*. The dietary composition and feeding rates were not significantly different between these two species, indicating a high degree of dietary overlap (see also Randall 1956; Feddern 1968; Fischer 1980). If there were inconsistencies in the dietary composition, combined with broadly nonoverlapping distributions of respective prey, this might prevent pairing and subsequent reproduction between these species.

*Chaetodon trifasciatus* and *C. lunulatus* were rare at Christmas Island and in both cases their abundances were one to three orders of magnitude lower compared to any other location for which abundance data are available (Adrim and Hutomo 1989; Findley and Findley 2001; Pratchett et al. 2004, 2006b; Pereira and Videira 2005). Rarity of one or both parental species plays a significant role in hybrid formation in reef fishes (Randall et al. 1977; Fisher 1980; Pyle and Randall 1994; Frisch and van Herwerden 2006; Yaakub et al. 2006; Marie et al. 2007; Maruska and Peyton 2007; Hobbs et al. 2009). Furthermore, one or both hybridizing species have been reported as rare in 11 (58%) of 19 locations where other butterflyfishes hybridize (Hobbs et al. In press).

*Chaetodon trifasciatus* and *C. lunulatus* form heterospecific pairs at Christmas Island indicating that assortative mating has broken down between these two sister species, probably as a consequence of their local rarity. Pairing in this species complex most likely occurs for reproduction, because *C. lunulatus* has been deemed a monogamous breeder (Yabuta 1997) and pair formation corresponds with the onset of sexual maturity (Pratchett et al. 2006a).

The nominal hybrids in this complex—initially identified through aberrant markings, intermediate to those of their parents (Hobbs et al. 2009)—had spatial and dietary ecologies comparable to those of their putative parents. Moreover, hybrids were observed in breeding pairs with both parent species, potentially facilitating gene transfer between these species—if hybrids are fertile. Importantly, hybrids in this complex were relatively rare and the statistical power associated with their data is therefore limited. Nevertheless, rarity of hybrids is, per se, interesting and might simply reflect the relative rarity of their parents, or be indicative of some selective pressure on hybrids.

### Genetics of hybridization

The use of both mtDNA and microsatellite loci allowed confirmation of the hybrid status of intermediately colored individuals, and shed light on the potential evolutionary consequences of hybridization in this complex. The mitochondrial cyt b identified two distinct parental clades, separated by 5% (29 fixed substitutions out of 522 nucleotides). The genetic distance between the species clades is consistent with divergence of these species, some 2.5–4 Ma (Bellwood et al. 2010), given the cyt b mutation rate of 1–2.5% Ma^−1^ (McMillan and Palumbi 1997) and assuming a molecular clock. Hybridization is most common between species that diverge by less than 10% because the genetic similarity increases the chance of viable hybrid formation (Mallet 2005). Each parental clade contained all individuals of the respective species, from all geographical locations. Hybrids shared cyt b haplotypes with the Pacific Ocean *C. lunulatus* only, indicating a unidirectional contribution to hybridization, in which *C. lunulatus* appears to always be the mother.

Despite informing about the matrilineal contribution, the results from mtDNA analyses were not sufficient to confirm the hybrid status of the intermediately colored individuals, which could simply be an aberrant color pattern of *C. lunulatus.* However, nuclear microsatellite markers ruled out this scenario and showed that hybrid genotypes were distinct from (and intermediate to) those of *C. trifasciatus* and *C. lunulatus*. This confirms the hybrid status and suggests that intermediately colored individuals were most likely F_1_ hybrids. The unidirectional maternal contribution contrasts with findings of other reef fish hybridization studies (McMillan et al. 1999; van Herwerden et al. 2006; Yaakub et al. 2006; Marie et al. 2007), which found that maternal contribution was either bidirectional or biased toward the more abundant species. This may suggest that hybrids resulting from the opposite cross (*C. trifasciatus* females) are subject to some negative selection or that female *C. lunulatus* are actively choosing (i.e., female choice, Wirtz 1999) to mate with *C. trifasciatus* males, because of lack of conspecifics. This latter explanation seems more likely because, even though abundances of the parental species in this complex were not statistically different, *C. lunulatus* was observed less frequently than *C. trifasciatus,* and in previous censuses of the ichthyofauna of Christmas Island it was not recorded (Allen et al. 2007; but see Hobbs et al. 2009; Hobbs et al. 2010).

The apparent lack of introgression between *C. trifasciatus* and *C. lunulatus* could be explained by the extreme rarity of the hybrids or their infertility. F_1_ hybrids of this complex sampled at Christmas Island previously to this study were sexually mature (J-P. Hobbs, unpublished data), henceforth the rarity of these individuals seems to be the most likely explanation. Three conditions are required for introgression to take place: (1) a fertile hybrid with the mtDNA of one species must (2) pair with an individual of the other species and (3) produce viable offspring (see Fig. 8 in Yaakub et al. 2006). These three conditions have to be sequentially satisfied frequently enough for the genetic signal of introgression to perpetuate and be detected. The hybrids in this complex may not be abundant enough to meet the conditions for introgression. In addition, *C. lunulatus* appears to be a recent colonist to Christmas Island (Hobbs et al. 2010) and there may not have been sufficient time for introgression to be evident in the genetic composition of these species. Lack of introgression contrasts with the results of most reef fish hybridization studies (van Herwerden and Doherty 2006—northern hybrid zone; van Herwerden et al. 2006; Yaakub et al. 2006; Marie et al. 2007; but see van Herwerden and Doherty 2006—southern hybrid zone; Yaakub et al. 2007) and, more importantly, with the findings of other studies of butterflyfish hybridization (McMillan et al. 1999).

The different divergence ages of parental species might account for the dissimilarities found in the outcome of hybridization in reef fishes. At the Solomon Islands, *C. punctatofasciatus* hybridizes with *C. pelewensis* (McMillan et al. 1999) from which it differs by only 0.7% of the mitochondrial cyt b (McMillan and Palumbi 1995). Bidirectional introgression between these species is so extensive that, within the hybrid zone, hybrid phenotypes account for over 70% of the individuals (McMillan et al. 1999). Moreover, despite phenotypic differentiation of the two species evident outside the hybrid zone, their genetic homogeneity extends for thousands of kilometers beyond the hybrid zone (McMillan et al. 1999) as a result of extensive bidirectional introgression. Conversely, taxa in the *C. trifasciatus* complex show a clear mtDNA break and hybridization between species is unidirectional and lacks introgression, with apparently more localized, limited evolutionary consequences. From the data available, it appears that increased genetic divergence between hybridizing butterflyfishes signifies a higher fitness cost to hybridization and limits introgression.

This is consistent with other reef fish hybridization studies in which the authors found that introgression was characteristic of more recently diverged taxa (Doherty et al. 1994; Planes and Doherty 1997a, b; van Herwerden and Doherty 2006; van Herwerden et al. 2006; Yaakub et al. 2006, 2007; Marie et al. 2007). For example in the Labridae, hybridization between *Thalassoma jansenii* and *T. quinquevittatum-*–for which divergence is dated at 5–10 Ma and genetic distance less than 2% at cyt b (Bernardi et al. 2004)—was bidirectional and introgressive (Yaakub et al. 2006), whereas in the relatively older species *Halichoeres garnoti* and *H. bivittatus* (11–18 Ma, genetic break >5.5% based on three mtDNA markers) (Barber and Bellwood 2005) hybridization was deemed to have limited evolutionary significance, due to the apparent lack of introgression and rarity of hybrids (Yaakub et al. 2007).

Similarly, the hybridizing color morphs of *Acanthochromis polyacanthus* in the northern hybrid zone on the Great Barrier Reef (GBR), not differentiated by allozyme data (Planes and Doherty 1997b), showed high levels of mtDNA Hypervariable Region 1 (HVR1) introgression (van Herwerden and Doherty 2006). In contrast, the hybridizing southern GBR *A. polyacanthus* color morph, divergent from all other populations by 2.8% mtDNA cyt b (Planes et al. 2001), showed a distinct allozyme structure (Planes and Doherty 1997a) and negligible introgression in the HVR1 (van Herwerden and Doherty 2006).

Further, introgression and bidirectional maternal contribution were found in hybridizing surgeonfishes *Acanthurus leucosternon* and *A. nigricans*, for which 1% genetic break was reported in the mtDNA COI marker used (Marie et al. 2007). In hybridizing groupers, *Plectropomus leopardus* and *P. maculatus,* separated by 1% genetic break based on two nuclear and two mtDNA markers (Craig and Hastings 2007), hybridization was highly introgressive, but the maternal contribution was unidirectional (van Herwerden et al. 2006). Based on the available data, it seems that for reef fishes a genetic break smaller than 2%—at a range of mtDNA loci other than HVR1—results in introgressive hybridization, which can have evolutionary consequences extending outside the hybrid zone and may result in increased genetic diversity and adaptability (e.g., McMillan et al. 1999). Conversely, a genetic break larger than 5%, albeit still lower than the proposed 10% cutoff for successful hybrid formation in the terrestrial environment (Mallet 2005), seems to result in a higher cost to hybridization and a reduction in the number of viable, fertile hybrids.

In addition to evolutionary relatedness, ecological factors may influence the occurrence of hybridization. Sustained pressure on coral reefs worldwide, which decreases available habitat and negatively affects the abundance of some reef fishes (e.g., Pratchett et al. 2006b; Graham 2007), might result in increased habitat overlap and local rarity of species—the ecological conditions most frequently ascribed a role conducive to hybridization in reef fishes. Further, ocean acidification negatively impacts mate recognition in reef fishes (Munday et al. 2009), increasing the chances of heterospecific breeding. While increased cases of hybridization between recently diverged reef fishes may prove beneficial to the adaptability of the species involved, a greater number of cases of hybridization among more distantly related species might have a detrimental effect and it may also result in reverse speciation (two species become one).

### Evolutionary consequences of hybridization

The genetic diversity of *C. trifasciatus* is relatively low within the hybrid zone. Low genetic diversity can have a detrimental effect on the adaptability of species to novel environments (Rhymer and Simberloff 1996), and in this case might result in the local extinction of *C. trifasciatus*. For this species, hybridization might represent a significant cost. Indicative of this is the apparent lack of maternal contribution to hybridization from *C. trifasciatus.* Female *C. trifasciatus* form pairs with *C. lunulatus* males (J-P. Hobbs, unpublished data) and it seems therefore possible that the resulting hybrids may not be viable, and heterospecific mating may represent a significant reproductive cost. Moreover, the hybrids of this complex that do survive (those with *C. lunulatus* mothers) have the lowest genetic diversity and, compared to their parents, may be less well adapted to the environmental conditions of Christmas Island (as hinted at by their more limited resource use and significantly lower abundance). These findings are in sharp contrast with those of the only previous genetic studies of hybridization in butterflyfishes (McMillan et al. 1999). McMillan et al. (1999) found that, within the hybrid zone at the Solomon Islands and Papua New Guinea, hybrid phenotypes largely outnumbered the pure *C. pelewensis* and *C. punctatofasciatus* suggesting better survival (and hence fitness) of hybrids than parents within the hybrid zone environment.

Hybridization between *C. trifasciatus* and *C. lunulatus* at Christmas Island could be rare and relatively recent (and this could explain the lack of introgression), but, protracted through time, this may facilitate the local persistence of at least one of these rare species (*C. lunulatus*). Moreover, this study detected high levels of intrabasin connectivity in the Indian Ocean, with populations of *C. trifasciatus* from locations as far apart as Zanzibar and Christmas Island showing no significant genetic differentiation. According to previous studies of reef fish hybridization (Yaakub et al. 2006; Marie et al. 2007; Hobbs et al. 2009), hybrid individuals could stray further west to Cocos (Keeling) Islands. However, *C. trifasciatus*×*C. lunulatus* hybrids seem to be restricted to the hybrid zone, with limited, more localized consequences, a scenario also found in Caribbean wrasses of genus *Halichoeres* (Yaakub et al. 2007).

## Conclusions

Hybridization between *C. trifasciatus* and *C. lunulatus* at Christmas Island is facilitated by a combination of similarities in habitat and resource use, low abundance, apparently random mating behavior, and limited genetic divergence. Hybrids resulting from at least one of the alternative possible crosses are viable. However, the apparent lack of introgression between *C. trifasciatus* and *C. lunulatus* and the rarity of the hybrids suggest that hybridization in this complex is rare and relatively recent, and so far has had limited evolutionary consequences. Hybridization between *C. trifasciatus* and *C. lunulatus* might facilitate persistence of at least one of these locally rare species at Christmas Island (*C. lunulatus*), while it may result in the local extinction of the Indian Ocean species *C. trifasciatus*, for which hybridization appears to represent a significant cost. Future research should be directed toward hybrid fitness in this complex to ascertain why hybrids are so rare and why crosses in which *C. trifasciatus* is the mother were not detected in the genetic analyses. Also, the hybridization scenario presented here warrants careful temporal monitoring of the Christmas Island hybrid zone, to determine whether hybridization between *C. trifasciatus* and *C. lunulatus* is indeed an isolated process or whether it is the start of the replacement of an Indian Ocean species by a Pacific Ocean species at this location.

In the face of potential future increased instances of hybridization in reef fishes, trends identified here should be further evaluated. The inclusion and similar characterization of other butterflyfish species pairs that hybridize could further help elucidate the relative importance of different factors in promoting hybridization and identify common trends in the evolutionary (genetic) outcomes. Clearly more studies are required to further elucidate the mechanisms conducive to hybridization in this group and also to determine the evolutionary consequences. Further, the inclusion of taxa belonging to different families could help explain the disproportionate incidence of hybridization in the Chaetodontidae and in reef fishes generally. Long-term monitoring of known hybrid zones (Arnold and Martin 2010), such as Christmas Island (Hobbs et al. 2009), including changes in coral reef health and abundance of live corals over time, could provide insights into the adaptive consequences of hybridization in a changing world.
